# Cardiac safety of the adjuvant Trastuzumab in a Moroccan population: observational monocentric study of about 100 patients

**DOI:** 10.1186/1756-0500-6-339

**Published:** 2013-08-28

**Authors:** Meryem Aitelhaj, Siham LKhouyaali, Ghizlane Rais, Amina Mohtaram, Soundouss Raissouni, Brahim Ghissassi, Saber Boutayeb, Hind Mrabti, Youssef Bensouda, Hassan Errihani

**Affiliations:** 1 Medical oncology Department, National Institute of Oncology, Rabat, Morocco

## Abstract

**Background:**

Trastuzumab is a humanized monoclonal antibody that binds to the extracellular domain of the human epidermal growth factor receptor 2 (HER 2) and inhibits carcinoma cellular proliferation. Its use as an adjuvant for a period of one year is currently an internationally recognised standard for the treatment of localized breast cancer. Its use is generally well tolerated, with the most salient side effect being a particular cardiotoxicity that is typically manifested by an asymptomatic decrease in the left ventricular ejection fraction (LVEF) requiring careful monitoring before and during treatment. To evaluate the cardiac safety of trastuzumab we conducted a retrospective observational study of patients with HER2-positive localized breast cancer treated with trastuzumab between May 2008 and May 2010 in Morocco.

**Findings:**

The study comprised of 100 patients. The average in LVEF before the start of trastuzumab was 70%, and at the end of treatment 66%, a decrease in absolute terms of 4%; this difference was statistically significant. 38% of the patients exhibited cardiotoxicity. 97% of our patients have completed treatment, of whom 23% with a provisional arrest because of a regressive fall in LVEF. A final arrest has been made in 3% of cases due to a non regressive reduction in LVEF. A symptomatic heart failure was found in three patients. Analysis of risk factors toxicity found a baseline LVEF higher in the patients who met cardiotoxicity than the rest of our sample.

**Conclusions:**

The cardiac safety in our study seems comparable with the literature data but located in the upper range of levels of toxicity. Cardiotoxicity is the major complication of Trastuzumab, of which LV dysfunction is the most common. Most instances are transient, asymptomatic and reversible.

## Introduction

Breast cancer is the most common malignant disease and among the most frequent causes of cancer mortality in females worldwide [1,2]. Overexpression of human epidermal growth factor receptor type 2 (HER2) occurs in 20-25% of invasive breast cancers, which is associated with a poor prognosis [3,4].

Trastuzumab is a humanized monoclonal antibody that binds to the extracellular domain of HER2 receptor and inhibits carcinoma cellular proliferation [5].

Trastuzumab therapy is important in the treatment of early and advanced disease as shown in multiple randomized trials. Its use as an adjuvant treatment for a period of 1 year is currently an international standard of care in HER 2 over expressed localized breast cancer. It is generally well tolerated, with a low incidence of adverse effects [6] of which the most relevant is cardiotoxicity that had not been anticipated on the basis of the results of preclinical or early clinical studies. It is typically manifested by an asymptomatic decrease in left ventricular ejection fraction (LVEF) and less often by clinical heart failure [7].

It requires careful monitoring of the LVEF before and during treatment. Trastuzumab cardiotoxicity was originally described in women with metastatic breast cancer and in several subsequent trials of adjuvant trastuzumab, about 80% of trials show cardiotoxicity [8]. However, the incidence of cardiotoxicity amongst populations of women treated outside of this clinical trial is not well known.

The purpose of this study is to evaluate the cardiotoxicity incidence rate associated with adjuvant trastuzumab treatment in clinical practice in a sample Moroccan population, by describing its characteristics, management and potential associated risk factors.

## Patients and methods

This is a retrospective observational institutional study conducted at the Department of Clinical Oncology, in the national institute of oncology of Rabat from May 2008 to May 2010.

### Eligibility criteria

Eligible patients had localized breast cancer verified histologically and HER2 positive status assessed by immunohistochemistry (3+) or fluorescent in situ hybridization positivity; adequate cardiac function with normal LVEF ≥ 50% measured on echocardiography and who received adjuvant Tarstuzumab.

Ineligibility criteria included a history of documented congestive heart failure, coronary artery disease with previous Q-wave myocardial infarction, angina pectoris requiring medication, uncontrolled hypertension, clinically significant valvular disease and unstable arrhythmias.

The study respected the ethical rules for medical research involving human subjects as stipulated by the World Medical Association in the Declaration of Helsinki. The local ethical committee of the national institute of oncology of Rabat also approved this study; and patients gave their consent.

Cardiac monitoring included physical examination and an assessment of LVEF by echocardiography: it was evaluated before Trastuzumab administration and every 12 weeks thereafter for the duration of therapy.

Cardiotoxicity was defined as a LVEF decrease below normal values (50%) or an absolute decrease of >10 points below the baseline value or any symptoms or signs of heart failure.

The following cardiovascular risk factors were analysed: age, overweight body mass index (BMI > 25 kg/m2 and < 30 kg/m2), obesity (BMI ≥ 30 kg/m2), hypertension, diabetes, LVEF at baseline, number of anthracycline cycles.

### Follow up

Patients were followed up until May, 2012. Any patients who were not reviewed in the last consultation were contacted again by telephone.

### Statistical analysis

Data was analysed using an electronic CRF (case report form).

The information was recorded in an Excel database and analysed with the statistics software SPSS, version 12.0. A logistical regression analysis was performed to examine which variables influenced whether or not a patient exhibited cardiotoxicity. A p value less than 0.05 was considered significant.

## Findings

A total of 100 patients were enrolled. The median age at diagnosis was 46 years [range: 24-74]. According to TNM staging, 11 patients had T1, 50 patients (50%) had T2 and 39% had T3 or T4. 71 patients (71%) had positive lymph nodes. 67% of our patients were hormone receptor positive.

All patients received surgery, 22% had conservative surgery and 78% received radical mastectomy with axillary lymph nodes dissection. 40 patients (40%) received chemotherapy based on anthracyclines and 60% had sequential chemotherapy with anthracyclines and taxanes. 93 patients had Radiotherapy, of whom 44 on the left breast. All patients received adjuvant Trastuzumab. The baseline characteristics of the patients and the Treatment Delivered are detailed in Table 1. It must be stated that before starting the Trastuzumab treatment, 11% of the patients exhibited arterial hypertension and 7% diabetes.

**Table 1:** Characteristics of patients and treatment delivered

**Characteristic**	
Age	46 [24-74]
menopausal Status	
postmenopausal	31%
Premenopausal	69%
Breast	
right	53%
Left	47%
Tumor size	
T1	11%
T2	50%
T3	26%
T4	13%
Ganglion involvement	
Positive	71%
Negative	29%
Hormone receptor status	
Positive	67%
Negative	33%
Surgery	
radical	78%
conservative	22%
Chemotherapy	
Anthracycline	40%
Taxanes (sequential)	60%
Radiotherapy	93%
Right breast	49%
Left breast	44%

The average in LVEF at baseline was 70% [55%-89%], and it was 66% [42%-87%] at the end of treatment, a decrease in absolute value by 4%. This difference was statistically significant with p < 0, 0001.

38% of the patients presented cardiotoxicity according to the predefined criteria; the details are listed in Table 2.

**Table 2:** Characteristics of the LVEF decrease in patients with cardiotoxicity (n = 38)

**Cardiotoxicity criteria**	**No. patients**	**% Patients with cardiotoxicity**	**% of patient total**
LVEF decrease <50%	7	18,42	7
LVEF decrease >10 points below baseline	16	42,10	16
LVEF decrease > 15 points below baseline	22	57,89	22

Of the 38 patients who presented cardiotoxicity, 23 (60.52%) had to discontinue treatment; this number constitutes 23% of the patient total. In 20 patients, treatment was resumed after normalising parameters, the mediane recovery time of LVEF was 21 days in both anthracycline alone and sequential chemotherapy; 9 of these cases received pharmacological treatment with angiotensin converting enzyme inhibitors/angiotensin receptor antagonists.

Treatment was completed in 97% of our patients. Definitive Trastuzumab interruption was necessary in 3 patients due to the non-recovery of the ventricular function. A good clinical improvement was reached under medical treatment. Three cases of grade 2 heart failure (HF) according to the New York Heart Association (NYHA) were noticed.

As shown in Table 3, when we analyse the influence of each separate predictive factor for cardiac dysfunction and the appearance of cardiotoxicity, we find no statistically significant differences for age, arterial hypertension, diabetes, obesity, number of anthracycline cycles or chemotherapy regimen (sequential/ anthracycline alone). Patients who experienced toxicity have a slightly higher mean age than those who did not, and relatively high prevalence of obesity (42.1%); Paradoxically LVFE at the baseline was significantly higher in the group of patients who developed cardiac complications.

**Table 3:** Risk factors in total population, in group with and without cardiac complications

**Risk factor**	**Total population**	**Group with cardiac complications**		**Group with no cardiac complications**		**Value of p no complications vs complications**
	**N = 100**	**N = 38**		**N = 62**		
Age (years)	46	47		45		0.96
Overweight	38%	12 (31.57%)		26 (41.93%)		0.61
obesity	35%	16 (42.10%)		19 (30.64%)		0.57
Hypertension	11%	4 (10.52%)		7 (11.29%)		0.88
Diabetes	7%	3 (7.89%)		4 (6.45%)		0.31
LVFE baseline	70%	74%		67%		< 0.001
Chemotherapy with or without anthracycline	100%	Sequential	25 (65.5%)	Sequential	35 (56.5%)	0.3
		anthracycline	13 (34.5%)	anthracycline	27 (43.5%)	
Nb of anthracycline cycles	4	4		4		0.61
Concomitant radiotherapy with trastuzumab	69	24 (63%)		45 (72%)		0.318

## Discussion

The resultant cardiotoxicity of the treatment in oncology often limits its benefits [9]. This is why monitoring and early prevention of aggressive anti-tumour treatment cardiac complications is of clinical interest. Trastuzumab is the standard drug for treating patients with breast cancer that over express HER2 with the most adverse effect being cardiotoxicity. It was initially described in women with metastatic breast cancer, with a higher incidence rate when trastuzumab was administered with anthracyclines [10]. Based on this data, the concomitant use of (anthracyclines and trastuzumab) was discouraged due to the greater risk of causing cardiotoxicity. Subsequently, several adjuvant clinical trials observed cardiotoxicity, which was considered acceptable [8-11]; Cardiac function was carefully monitored, but difference exists among the trials and how cardiotoxicity was defined. In general these trials demonstrated that the risk of symptomatic congestive heart failure was low; but reveal a much greater asymptomatic decreases in left ventricular ejection fraction [12].

However, the cardiotoxicity incidence rate in the population of women receiving treatment outside of clinical trials is unknown. The current study provides insight into the common experience of trastuzumab use in real life situations common in oncology and cardiology clinics. In a retrospectively evaluated population of 100 HER2 positive, early and locally advanced breast cancer women treated with trastuzumab in the adjuvant setting, 38% of the patients exhibited cardiotoxicity and nearly 23% required discontinuation of the medication due to cardiac complications.

The incidence of left ventricular dysfunction in our study (38%) is consistent with that reported in the combined analysis of National surgical adjuvant breast and bowel project (NSABP) B-31 (34%) [13], less than in Breast Cancer International Research Group (BCIRG) 006 trial (18%) [14] and higher than in the HERceptin Adjuvant (HERA) trial (7.1%) [15]. In our study, 23% of patients had to suspend trastuzumab treatment, whether temporarily or definitively, due to cardiotoxicity. In the McArthur and Chia study [16] the rate of treatment suspension due to cardiac dysfunction was 21.6% which is higher than the number reported in the HERA clinical trial [17] and closer to the rate shown by our patient’s series. However, the same study shows that most of the patients who discontinued treatment were able to restart it after recovering their cardiac function; in our study 20 of the patients who interrupted treatment ended up suspending it definitively.

Symptomatic heart failure events have been observed much less frequently than asymptomatic LV dysfunction. 1.9% in BCIRG 006, 4% in NSABP B-31 and 0.6% in the HERA trial of trastuzumab treated patients reported severe symptoms of HF (NYHA III/IV) [13-15]. Tarantini et al. reported 3% symptomatic heart failure incidence in a cohort of 499 women with HER2 positive early breast cancer from 10 Italian institutions treated with trastuzumab observed retrospectively [18]. Such incidence is consistent with that in our study, in which 3% of patients complained of mild heart failure symptoms.

The majority of complications are asymptomatic. The asymptomatic nature of cardiac complications makes monitoring of trastuzumab therapy safety mandatory. This issue has been addressed and regulated by a few guidelines [19,20]. They suggest that detection of heart damage due to trastuzumab therapy is best accomplished via sequential measurements of LVEF, either by multiple-gated acquisition scans (MUGA) or by echocardiography techniques. In addition to imaging methods, the literature suggests that measurement of plasma markers, such as brain natriuretic peptide (BNP) as a marker of LV stretch and cardiac troponins as markers of myocardium disintegration, may be used to predict and to detect cardiac dysfunction during treatment with trastuzumab [21].

We found no differences in prevalence of cardiovascular risk factors between the group of patients with and without cardiac complications; excepted LVEF at baseline, it was paradoxically higher in the group of patients who developed cardiotoxicity. The association of cardiovascular risk factors with trastuzumab related cardiotoxicity is not apparent and not fully explained. The most well-known independent risk factors are advanced age and previous exposure to anthracyclines [22]. In the NSABP B-31 and NCTTG N9831 trials age ≥ 50 years, requirement for hypertension medications, previous exposure to anthracyclines and LVEF at baseline < 55% were risk factors for heart failure in the course of trastuzumab treatment in univariate analysis [23,24]. In the HERA trial overweight and obesity were risk factors for cardiac toxicity but age, hypertension, dyslipidaemia and previous heart disease did not increase the risk of trastuzumab-related cardiotoxicity [15]. In a large retrospective cohort study the authors observed that anthracycline and Trastuzumab were associated with increased HF [25].

The mechanisms of trastuzumab related cardiotoxicity remain uncertain. Genetic background has been suggested by some studies [26,27]. There are two different hypothesis of trastuzumab mechanism of cardiac damage in the literature. Some preclinical data indicate that inhibition of the myocardial HER2 receptor leads to changes in the tertiary structure of the cardiac contractile apparatus, which seems likely to be a reversible effect [28]. Others suggest that trastuzumab induces apoptosis and cardiomyocytes’ death, which is likely a progressive and rather irreversible condition [29]. Both mechanisms may play a role in cardiotoxicity and numerous external factors may decide which predominates and whether heart damage is reversible.

The cardiotoxicity of trastuzumab is always reversible (87% in our study) such as reported in others retrospectives studies in adjuvant and metastatic setting [23,30].

The limitations of our study are its small sample size and short follow-up. Despite these limitations, the results we obtained coincide with published studies, in that trastuzumab related cardiotoxicity in a clinical care setting is more frequent than is shown by clinical trial estimates.

## Conclusion

In conclusion, for trastuzumab, cardiotoxicity is its major complication with LV dysfunction being the most common case. Most incidences are transient, asymptomatic and reversible. Nevertheless, longer follow-up is needed to confirm that cardiotoxicity associated with trastuzumab therapy does not affect it’s long-term outcome. Prior to the institution of trastuzumab therapy, all patients should be evaluated for their cardiovascular status. As the majority of complications are asymptomatic, routine cardiac monitoring must be performed during trastuzumab treatment.

## Abbreviations

HER2: Human epidermal growth factor receptor 2; LVEF: Left ventricular ejection function; HF: Heart failure; NCI-CTC: National Cancer Institute Common Toxicity Criteria; BMI: Body mass index; CRF: Case report form.

## Competing interests

The authors declare that they have no competing interests.

## Authors’ contributions

MA, SL and GR were involved in the analysis of the data and the literature research and they also wrote the manuscript. AM, SR and SB helped with the literature research. HM and YB helped with the literature research. YB helped with modifications and revision of the manuscript. HE approved the treatment and analyzed the literature data. All authors read and approved the final manuscript.
